# Tumor-homing exosomes enable targeted delivery of siRNA and isoimperatorin for overcoming BTK inhibitor resistance in DLBCL

**DOI:** 10.1016/j.mtbio.2025.102267

**Published:** 2025-09-02

**Authors:** Ruowen Sun, Yanchao Yang, Bin Zhang, Jiayuan Chen, Ye Wang, Zehui Jiang, Linlin Zhang, Milad Ashrafizadeh, Gautam Sethi, Jing Shen, Zuofei Chi

**Affiliations:** a The Second Department of Pediatric Hematology, Shengjing Hospital of China Medical University, Shenyang, 110004, Liaoning, China; b Department of Anesthesiology, Shengjing Hospital of China Medical University, Shenyang, 110004, Liaoning, China; c The First Department of Pediatric Hematology, Shengjing Hospital of China Medical University, Shenyang, 110004, Liaoning, China; d Department of Radiation Oncology and Shandong Provincial Key Laboratory of Radiation Oncology, Shandong Cancer Hospital and Institute, Shandong First Medical University and Shandong Academy of Medical Sciences, Jinan, Shandong, 250117, China; e Department of Pharmacology and NUS Centre for Cancer Research, Yong Loo Lin School of Medicine, National University of Singapore, Singapore, 117600, Singapore; f The Second Department of Hematology, Shengjing Hospital of China Medical University, Shenyang, 110004, Liaoning, China

**Keywords:** Non-hodgkin lymphoma, B cell lymphoma, Drug delivery system, Exosome, BTK, Isoimperatorin

## Abstract

Diffuse large B-cell lymphoma (DLBCL) is the most common type of lymphoma, but over one-third of patients relapse or develop refractory disease after first-line therapy. Novel therapeutic strategies are required to address persistent unmet clinical needs for DLBCL. This study aimed to develop an exosome-based drug delivery system for the targeted combination therapy of siRNA against Bruton's tyrosine kinase (BTK, an established therapeutic target in B cell lymphomas) and isoimperatorin (ISOIM, an active natural furanocoumarin showing anti-tumor effects) in DLBCL. Tumor exosomes were isolated as the delivery carrier. ISOIM/siBTK@Exosome was prepared by encapsulating ISOIM and si-BTK into exosome using electroporation. Cellular uptake, immune escape, targeted delivery efficiency, anti-lymphoma activity and biosafety of ISOIM/siBTK@Exosome were evaluated in two DLBCL cell lines and in tumor-bearing mice. ISOIM/siBTK@Exosome displayed significant anti-lymphoma activity compared to ISOIM@Exosome or siBTK@Exosome alone, demonstrating synergistic therapeutic role of ISOIM and si-BTK. Besides, ISOIM/siBTK@Exosome can accelerate T cells activation and prevent macrophage M2 polarization in vitro. Administration of ISOIM/siBTK@Exosome to tumor-bearing mice significantly inhibited tumor growth and prolonged survival. The ISOIM/siBTK@Exosome was biocompatible and biosafe in vivo without damage on the major organs in H&E staining. The prepared ISOIM/siBTK@Exosome may provide novel targeted therapeutic strategy to be applied in the clinical management of patients with DLBCL.

## Highlights

• ISOIM/siBTK@Exosome is a highly effective and non-toxic therapeutic strategy in DLBCL.
• Targeted delivery of si-BTK overcomes the acquired resistance to current BTK inhibitors and toxicity.
• ISOIM and si-BTK showed synergistic therapeutic effect in DLBCL.
• ISOIM/siBTK@Exosome could promote T cells activation and prevent macrophage M2 polarization.

## Graphical abstract

**Figure:**
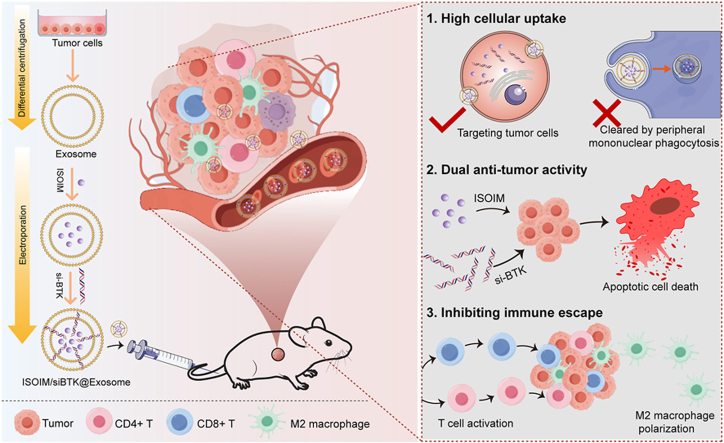

## 1 Introduction

Lymphoma is a general term for a group of immune system malignancies originating in lymph nodes and lymphoid tissue, with more than 90 subtypes. Lymphoma is traditionally divided into Hodgkin and non-Hodgkin lymphoma (NHL), with NHL accounting for about 90 % of all lymphomas [1]. Global cancer statistics estimate approximately 553,010 newly diagnosed NHL cases and 250,475 deaths caused by NHL in 2022 [2]. China bears responsibility for approximately 20.1 % of these new cases and 17.4 % of deaths worldwide [3]. Diffuse large-B-cell lymphoma (DLBCL) is the most common type of NHL, with only 35–40 % patients can be cured with anthracycline-based chemotherapy because that DLBCL is a group of diseases that are heterogeneous in morphology, genetics, clinical presentation, and responsiveness to chemotherapy [[4], [5], [6], [7], [8]]. Therefore, seeking new treatment strategies can provide more opportunities for improving the clinical treatment of this disease.

Abnormal activation of the B cell receptor (BCR) signaling pathway is common in a variety of B-cell malignancies [9,10]. Bruton's tyrosine kinase (BTK) is a key enzyme in the BCR pathway and plays a key role in almost all aspects of B cell development, such as proliferation, activation and apoptosis [11]. BTK is activated by the upstream molecule spleen tyrosine kinase (SYK) and activates downstream phospholipase Cγ2, which ultimately promotes nuclear factor κB (NF-κb) nuclear translocation leads to B-cell hyperproliferation, malignancy, and inhibition of apoptosis, thus leading to various lymphomas [11], including DLBCL. BTK has been established as an important therapeutic target for B-cell lymphoma, and several BTK inhibitors have been developed, which show great therapeutic potential in B-cell malignancies both in preclinical and clinical studies [[12], [13], [14]]. However, its use as a monotherapy is limited, and there exists off-target effects of these inhibitors [15]. With the use of the drug, some patients have developed primary or secondary resistance to BTK inhibitors [16]. Hence, developing a new strategy that particularly targets BTK is considered more hopeful therapy. Small interfering RNAs (siRNA) has emerged as an attractive targeted therapy strategy for a variety of diseases due to its specific sequences designed to silence targeted genes, such as specific cancer-related genes [17,18]. However, challenges including short half-life, poor blood circulation, unsatisfactory membrane penetration, and low stability limit the use of free siRNA for clinical treatment. More information on siRNA delivery can be found in these studies [19,20].

Exosomes are nanoscale (30–150 nm) extracellular vesicles secreted by a variety of cells, and exhibits outstanding biocompatibility, targeting and stability in vivo [21]. Given this, exosome has attracted more and more attention as drug delivery carrier, especially suitable for the delivery of protein, nucleic acid, gene therapy agents [22,23]. Besides, exosomes can be used as a medium to realize the combination of multiple treatment modalities [24,25]. Natural Chinese herbal medicine has proven to be a valuable source for anti-tumor drugs screening [26]. Isoimperatorin (ISOIM), an active natural furanocoumarin, mainly distributes in herbal medicines Radix *Notopterygii*, Angelica *dahurica*, Radix *Aristophanae*, and coumarin. ISOIM possesses multiple pharmacological properties, including anti-inflammatory, anti-viral, inhibiting angiogenesis and other pharmacological effects [[27], [28], [29]]. Besides, ISOIM also exhibits anti-tumor activity several human cancers [[30], [31], [32]]. For example, ISOIM could inhibit cell viability and promote the apoptosis of hepatocellular carcinomas cells by inhibiting c-Myc and SIRT1 signaling axis [33]. Considering that low molecular weight compounds are easily excreted via glomerular filtration or liver metabolism, Zhao et al. constructed a nano-platform to load ISOIM, and this nano-platform showed significant therapeutic effect in NHL, mainly manifested as blocking cell cycle and facilitating mitochondrial-mediated apoptosis [34].

**Fig. 1:**
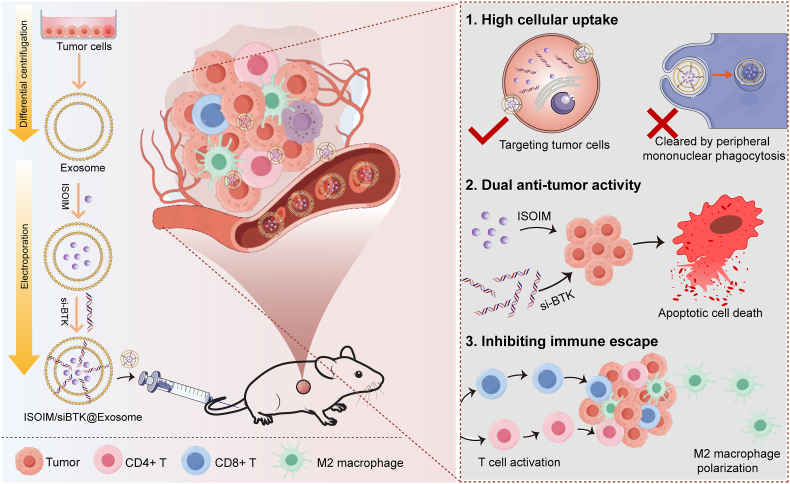
Scheme of the prepared ISOIM/siBTK@Exosome for DLBCL therapy.

Herein, to achieve dual anti-tumor effects, we proposed an exosome-targeted delivery strategy for the co-delivery of ISOIM and BTK siRNA (si-BTK) to tumor cells. As illustrated in Fig. 1, we first isolated exosomes from the DLBCL tumor cells, and then encapsulated the si-BTK and ISOIM into exosomes by means of electroporation. The prepared ISOIM/siBTK@Exosome possessed the homing properties inherited from exosomes and could target its parent cells, thus efficiently delivering the loaded si-BTK and ISOIM to lymphoma cells.

## 2 Materials and methods

### 2.1 Cell lines

Human B Lymphoma cell line SU-DHL-4 (#CRL-2957, ATCC, USA) and OCL-Ly8 (#JLC-G14773, Gelatins biological reagent Co., LTD, Jiangxi, China) were cultured in the RPMI-1640 medium with an additional adding of 10 % FBS, penicillin (100 U/mL) and streptomycin (100 μg/mL) in an incubator of 5 % CO_2_ at 37 °C.

### 2.2 Animals

BALB/c mice (six-week-old, male) were fed in a SPF environment with free access for water and food. All animal experiments were approved by the Institutional Animal Care and Use Committee of YANGZHOU UNIVERSITY (Approval No. 202410028), and conducted in accordance with ARRIVE guidelines.

### 2.3 Isolation of tumor-derived exosomes

To isolate exosomes from tumor cells SU-DHL-4, the SU-DHL-4 cells were cultured in RPMI-1640 medium as described in cell lines section, with the exosome-free FBS instead of the 10 % FBS. The exosome-free FBS was prepared by ultracentrifugation at 120,000 g and 4 °C for 18 h, followed by filtration with 0.22 μm syringe filters. Cells were grown to 80 % confluence, and the cells media were collected for differential centrifugation to remove dead cells (800 g for 5 min), cellular debris (1500 g for 15 min) and large extracellular vesicles (15,000 g for 30 min). Afterwards, the remaining supernatant were exposed to ultracentrifugation at 120,000 g and 4 °C to collect exosomes.

### 2.4 Preparation of ISOIM/siBTK@Exosome

The ISOIM and si-BTK-loaded exosomes, that is, ISOIM/siBTK@Exosome, were prepared by means of electroporation. Briefly, ISOIM (2 mg) and Exosomes (1 × 10^9^, measured by NTA) were mixed in 200 μL electroporation buffer (1.15 mM KH2PO4, pH 7.2, 25 mM KCl and 21 % Optiprep) for electroporation at 350 V, 150 μF and ∞Ω in electroporation cuvettes using an Electroporation System. Afterwards, the mixture was incubated at 37 °C for 30 min to allow the recovery of the exosome membrane. To remove the unbound ISOIM, exosomes were washed by pre-cold PBS and centrifuged at 4 °C (100,000×*g*). Then, the si-BTK (1.5 μg) was mixed with ISOIM-loaded Exosomes in electroporation buffer without RNase for loading si-BTK by electroporation, and the other conditions were the same as above. Afterwards, the mixture was then immediately placed on ice, and the exosomes were then treated with RNase to eliminate any siRNA attached to the exosome surface. The sequences of si-BTK are as follows: 5′-AAGUCAUACUCAUAGUAGGAG-3’ (sense), and 5′-CCUACUAUGAGUAUGACUUUG-3’ (antisense).

### 2.5 Characterization of ISOIM/siBTK@Exosome

The morphology of the isolated exosome and the prepared ISOIM/siBTK@Exosome nanoparticles were observed under transmission electron microscope (TEM) with voltage setting as 100 kV. In short, 10 μL samples diluted solution was placed on copper grid, stained with 2 % uranyl acetate and dried before TEM analysis. With the aids of a Malvern Zetasizer Nano ZS90, we evaluated the particle size distribution and zeta-potential of nanoparticles. Each sample was dispersed in deionized water before measurements. Expression of exosomes markers, CD63, CD81, TSG101 and calnexin, onto the isolated exosomes and the prepared ISOIM/siBTK@Exosome nanoparticles were detected utilizing western blot. To confirm the successful loading of si-BTK by the exosome nanoparticles, fluorescence co-localization of FAM-labeled si-BTK with the PKH26-labeled exosome was observed at confocal laser scanning microscopy (CLSM). To test the stability of ISOIM/siBTK@Exosome, which was dissolved in PBS (pH 7.4) or 10 % FBS-contained medium to observe the particle size and the polydispersity index (PDI) every 12 h, for a total of 14 times. To determine the loading efficiency of si-BTK loaded into ISOIM/siBTK@Exosome, a fluorescence microplate was employed to measure the content of FAM-labeled si-BTK at 488 nm, and the fluorescent signal intensity was calculated based on a pre-established standard curve. The loading efficiency of ISOIM into ISOIM/siBTK@Exosome was calculated based on the content of free ISOIM measured under ultraviolet spectroscopy at 230 nm. Release of ISOIM and si-BTK from the ISOIM/siBTK@Exosome was assessed under pH 5.5 and 7.4 in vitro. For this purpose, we dissolved the ISOIM/siBTK@Exosome into dialysis bags (cut-off 12 kDa) filled with 2 mL PBS, which was then soaked in the beaker bearing 20 mL of PBS (pH 5.5 and 7.4) away from light at 37 °C following sealing. Subsequently, at diverse time points, 1 mL of solution was collected for determining the content of ISOIM and si-BTK in PBS by using ultraviolet spectroscopy at 230 nm and 488 nm, respectively, and the same volume of fresh PBS with the same condition was replaced to the beaker. We calculated the release rate of ISOIM and si-BTK [ released content (OD of ISOIM and si-BTK in PBS)/the loaded content of ISOIM and si-BTK, respectively].

### 2.6 Cellular uptake assay

Uptake of ISOIM/siBTK@Exosome by SU-DHL-4 and OCL-Ly8 cells was studied by CLSM and flow cytometry (FCM). ISOIM/siBTK@Exosomes were labeled with PKH26, and then was added to SU-DHL-4 and OCL-Ly8 cells that seeded in confocal dishes for 6 h of incubation. Afterwards, cells were fixed and then were stained by DAPI for nucleus, followed by fluorescence imaging under a TCS_SP8 CLSM (Leica, Germany). For FCM analysis, cells were seeded into 6-well plate (5 × 10^5^/well), and then were co-incubated with PHK26-labeled ISOIM/siBTK@Exosomes after cells attachment. At several incubation time points, cells were collected and then were resuspended in PBS for FCM measurement. Additionally, to illustrate the uptake mechanism, we pretreated SU-DHL-4 and OCL-Ly8 cells with colchicine (5 μg/mL, #S18047, Yuanye, Shanghai), nystatin (15 μg/mL, #T25277, Yuanye) or chlorpromazine (10 μg/mL, #T92264, Yuanye) for 1 h, respectively. Afterwards, cells were co-incubated with PHK26-labeled ISOIM/siBTK@Exosomes for FCM measurement as described above.

### 2.7 Anti-lymphoma efficacy in vitro

SU-DHL-4 and OCL-Ly8 cells were seeded in 6-well or 96-well plates for preincubate. Then, cells were treated with exosome, free ISOIM, ISOIM@Exosome, siBTK@Exosome, or ISOIM/siBTK@Exosome for 24 h, respectively, to evaluate the anti-lymphoma efficacy of ISOIM/siBTK@Exosome, which was conducted by CCK-8, colony formation, Annexin-V/PI, live/dead staining and Transwell assays. For CCK-8 assay, 10 μL of CCK-8 solution was added for each well for another 2h of culture, and absorbance at 450 nm was measured to evaluate the cells viability. For colony formation, cells were cultured for 14 days after treatment. Then, colonies were washed with PBS, fixed with absolute methanol, and stained with crystal violet, followed by imaging and counting colony numbers. Calcein-AM/PI kit was employed for live/dead staining assay. In brief, cells were collected and resuspended (1 × 10^5^ cells/mL) in Calcein AM/PI staining solution. Following 15 min of incubation in the dark, survival status of cells was detected with the aids of a fluorescence microscope (EX/EM = 494/517 nm for alive cells in green; and EX/EM = 535/617 nm for dead cells in red). Annexin-V/PI assay was employed for detecting apoptosis. Briefly, cells were collected and resuspended in binding buffer. After staining by Annexin V-FITC and propidium iodide, cell apoptosis was determined by FCM. Transwell assay was employed for evaluating cell migration. Briefly, cells and 20 % FBS-contained complete medium were added to the upper and lower chamber of Transwell, respectively. Following 24h of incubation, cells entered to the lower chamber were fixed with absolute methanol, and stained with crystal violet, and were counted under the microscope (three randomly selected fields) with the aids of Image J.

### 2.8 qRT-PCR

Expression of BTK after ISOIM/siBTK@Exosome treatments was detected by qRT-PCR. Specifically, we first isolated the total RNAs to reversely transcribe it into cDNA. Next, PCR amplification was conducted with the aids of SYBR Green PCR Master Mix. The used primers are: BTK-F: 5′-GGTGGAGAGCACGAGATAAA-3’; BTK-R: 5′-CCGAGTCATGTGTTTGGAATAC-3’; GAPDH-F: 5′-AGCTTCAGCCCCAGGAAATC-3’; GAPDH-R: 5′-GACATACTGCTGGGCCAGTT-3’.

### 2.9 Western blot

Western blot was conducted to determine the protein expression of BTK, apoptotic markers (Bcl-2 and Bax) and exosomes markers (CD63, CD81, TSG101 and calnexin). Briefly, the isolated total proteins were separated by SDS-PAGE. After transferring proteins onto PVDF membranes, incubation with primary antibodies (BTK, #ab208937; Bcl-2, #ab182858; Bax, #ab32503; CD63, #ab134045; CD81, #ab155760; TSG101, #ab133586; calnexin, #ab22595; Abcam, Cambridge, United Kingdom) was carried out overnight at 4 °C, followed by secondary antibody incubation in the dark for 60 min. Finally, the blotting bands were visualized by means of ECL reagent followed by film exposure.

### 2.10 T‐cell activation and macrophage polarization

Human peripheral blood mononuclear cells (PBMCs) were obtained from Shanghai Bohu Biological Technology Co., Ltd.,China. CD3^+^ T lymphocytes in PBMCs were isolated with the aids of MACS CD3^+^ T cell Isolation Kit. CD3^+^ T cells were stimulated by cell stimulation cocktail (eBioscience™), and then were co-incubated with treated SU-DHL-4 cells (Exosome, ISOIM@Exosome, siBTK@Exosome, and ISOIM/siBTK@Exosome treatments, respectively) for 24 h. Subsequently, T cells were collected, followed by staining by CD69‐FITC, CD4‐PE‐CY7 and CD8‐PE. After fixation and permeabilization, T cells activation was measured by determining the proportion of CD69^+^CD4^+^ T cells and/or CD69^+^CD8^+^ T cells by FCM analysis.

For macrophage polarization, peritoneal macrophages were collected from mice, and were treated by IL-4 (15 μmol/L) and IL-10 (10 μmol) for inducing polarization towards M2 phenotype. Afterwards, macrophages were co-incubated with exosome or ISOIM@Exosome or siBTK@Exosome or ISOIM/siBTK@Exosome-treated SU-DHL-4 cells. Following 24 h of incubation, macrophages were collected, and stained by F4/80-PE and CD206-FITC, and were fixed and permeabilized for FCM analysis.

### 2.11 Anti-lymphoma efficacy in vivo

For subcutaneous xenografts, OCL-Ly8 cells (5 × 10^7^ cells) suspended in 100 PBS μL were injected subcutaneously to the right armpit of BALB/c mice. Mice were categorized into six groups for treatments when tumor volume reached 100 mm^3^, including control (PBS), Exosome, ISOIM, ISOIM@Exosome, siBTK@Exosome and ISOIM/siBTK@Exosome groups. For treatments, the corresponding drugs (at a dose of 20 mg/kg, ISOIM-equiv) were dissolved into PBS and were tail intravenously administrated to mice, and the in vivo anti-lymphoma efficacy was assessed by monitoring the weight and tumor volume of mice. Treatments lasted two weeks, and mice was then sacrificed to collect tumor and major organs tissues for histological staining.

### 2.12 Tissue biodistribution of ISOIM/siBTK@Exosome

DIR fluorescent probes-based in vivo imaging study was carried out to investigate the tissue distribution of the ISOIM/siBTK@Exosome. To be specific, ISOIM/siBTK@Exosome was labeled by DIR and then were tail intravenously administrated to OCL-Ly8 cells tumor-bearing mice (20 mg/kg), and the fluorescence intensity was monitored with the aids of an in vivo FX Pro imaging system. Moreover, ex vivo fluorescence imaging for major organ and tumor tissue was carried out following 48 h of injection.

### 2.13 Histological staining

For histological staining, the tissues were fixed by 4 % paraformaldehyde, embedded in paraffin, and were prepared into 5 μm sections for TUNEL staining and H&E staining, which were conducted for assessing apoptosis in tumor tissue and the potential toxicity of ISOIM/siBTK@Exosome to major organs. Meanwhile, expression of Ki67 and BTK in tumor tissue was detected by means of immunohistochemical staining. In short, tissue sections were exposed to citric acid and 3 % H_2_O_2_ for antigen retrieval and blocking, respectively. Then, sections were incubated with anti-Ki67 (#AF0198, Affinity) and anti-BTK (#ab208937, abcam) overnight at 4 °C and secondary antibody for 15 min, followed by examination under an optical microscope after DAB staining and hematoxylin re-staining.

### 2.14 Data statistics

All data was presented as mean ± SD, and were statistically graphed by GraphPad 7.0 software. For comparison between two groups or among three or more groups was conducted by unpaired *t*-test or ANOVA coupled with Tukey's post hoc test, respectively. P < 0.05 donates statistically significant.

## 3 Results

### 3.1 Construction and characterization of ISOIM/siBTK@Exosome

**Fig. 2:**
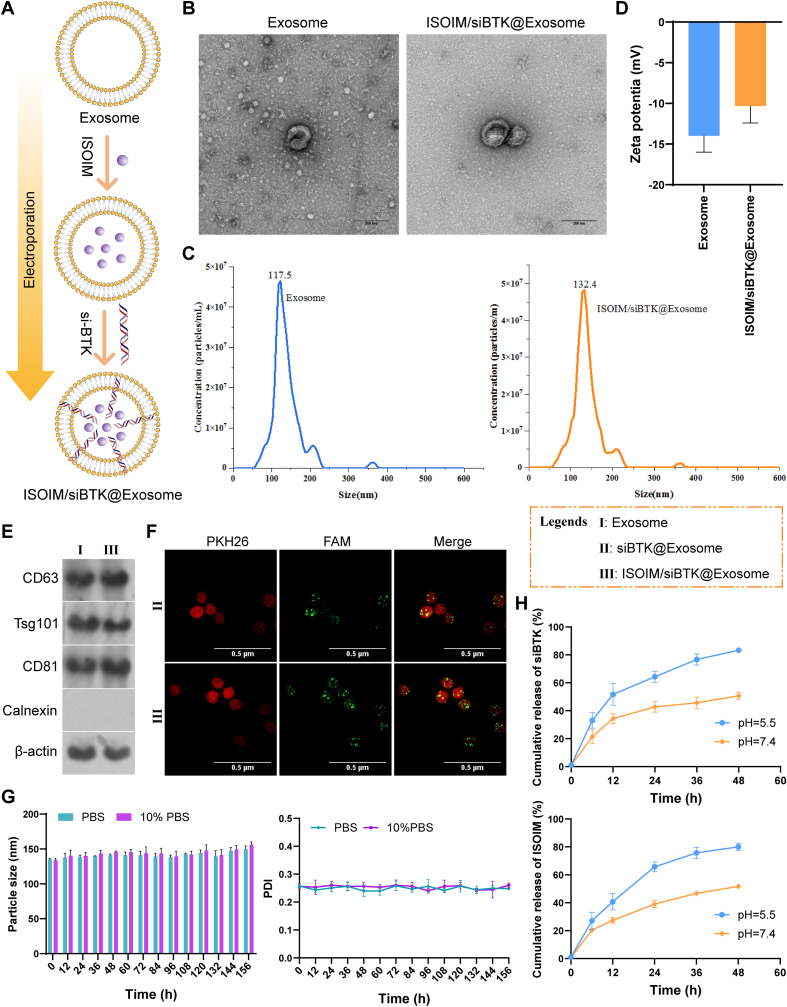
Preparation and characterization of ISOIM/siBTK@Exosome. A, Preparation process of the delivery system for ISOIM and siBTK based on exosome; B, Morphological characterization of empty exosome and ISOIM/siBTK@Exosome by means of TEM; C, Size distribution of empty exosome and ISOIM/siBTK@Exosome; D, Zeta-potential of empty exosome and ISOIM/siBTK@Exosome; E, Western blot showing the expression of exosome markers, CD63, CD81 and TSG101 in empty exosome and ISOIM/siBTK@Exosome; F, Confocal imaging showing the co-localization of PKH26-exosome and FAM-siBTK; G, Evaluation of stability by monitoring the variations of particle size and PDI of ISOIM/siBTK@Exosome in PBS and 10 % FBS medium; H, Release profiles of ISOIM/siBTK@Exosome at pH 5.5 and 7.4.

The preparation of ISOIM/siBTK@Exosome dual delivery system was shown in Fig. 2A. First, exosomes were isolated from the tumor cells by differential centrifugation, followed by loading of ISOIM and si-BTK into exosomes by electroporation. The morphology of empty exosomes exhibited typical “saucer-like” shape appearances, and similar shape appearances of ISOIM/siBTK@Exosome in TEM suggested that ISOIM and si-BTK electroporation had no significant effects on the morphology of exosomes (Fig. 2B). The peak particle size of empty exosomes was 117.5 nm, which was slightly increased to 132.4 nm when loading ISOIM and si-BTK into the exosomes (Fig. 2C). The zeta-potential (Fig. 2D) of empty exosomes were negatively charged (about −14 mV), which was slightly altered for ISOIM/siBTK@Exosome (about −10 mV), probably due to the positive charge of ISOIM. Western blot confirmed the enrichment of typical exosome markers, CD63, CD81 and TSG101 in both empty exosomes and ISOIM/siBTK@Exosome (Fig. 2E). According to the standard curve of ISOIM and ultraviolet spectroscopy at 230 nm, the loading capacity of ISOIM in exosomes was determined to be approximately 13.01 %. CLSM analysis revealed that the green fluorescence of FAM-labeled si-BTK was overlapped with the red fluorescence of PKH26-labeled exosomes, suggesting the effective loading of si-BTK in ISOIM/siBTK@Exosome (Fig. 2F). Based on the fluorescence of FAM-labeled si-BTK at 488 nm and corresponding standard curve, the loading capacity of si-BTK in exosomes was determined to be approximately 11.23 %. Altogether, the above findings indicated the feasibility of exosomes for loading ISOIM and si-BTK without affecting the physicochemical properties of the exosomes. During the 14 days of stability testing, the particle size and PDI showed no obvious fluctuations (Fig. 2G), implying an outstanding stability of ISOIM/siBTK@Exosome in vitro. The release profile of ISOIM and si-BTK from ISOIM/siBTK@Exosome was further captured at the pH conditions of 5.5 and 7.4 (Fig. 2H). Under acidic conditions (pH 5.5), the release rate of ISOIM and si-BTK reached about 80 % at 48 h, which was higher than that under the physiological conditions (pH 7.4).

### 3.2 Cellular uptake and tumor-targeting ability of ISOIM/siBTK@Exosome

**Fig. 3:**
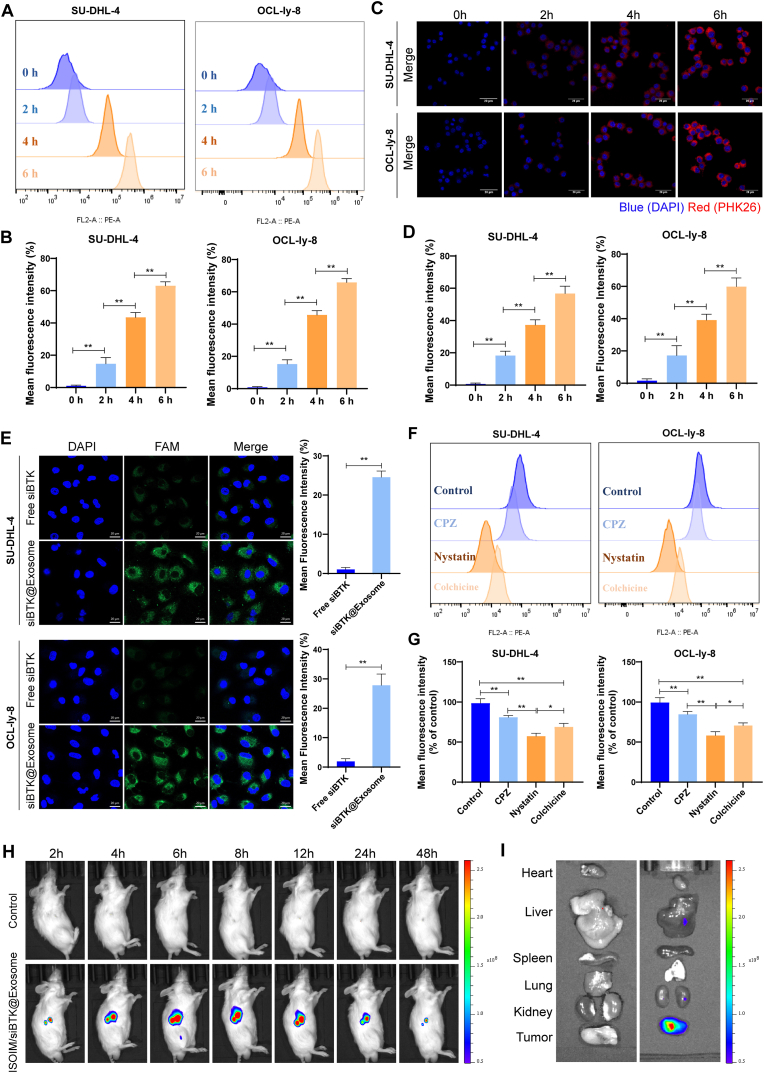
Cellular uptake and tumor-targeting ability of ISOIM/siBTK@Exosome. A-B, Flow cytometry results (A) and corresponding quantitative analysis (B) for evaluating the uptake of ISOIM/siBTK@Exosome by two lymphoma cell lines; C-D, CLSM images (C) and corresponding quantitative analysis (D) for evaluating the uptake of ISOIM/siBTK@Exosome by two lymphoma cell lines; E, CLSM images and corresponding quantitative analysis for evaluating the uptake of siBTK and siBTK@Exosome by two lymphoma cell lines; F-G, Flow cytometry results (F) and corresponding quantitative analysis (G) for evaluating the uptake mechanism of ISOIM/siBTK@Exosome by lymphoma cells; H, Tumor targeting of ISOIM/siBTK@Exosome assessed by in vivo fluorescence imaging; I, the ex vivo fluorescence imaging of the isolated major organ and tumor tissue at 48 h following injection of ISOIM/siBTK@Exosome. ∗P < 0.05; ∗∗P < 0.01.

In vitro cellular uptake of ISOIM/siBTK@Exosome by lymphoma cells was evaluated. The ISOIM/siBTK@Exosomes were labeled with PKH26, and then was added to SU-DHL-4 and OCL-Ly8 cells. FCM analysis indicated that fluorescence signals were enhanced gradually along with the incubation time in both cells, with fluorescence intensity over 60 % after 6 h of incubation (Fig. 3A and B). Similar results were also observed in CLSM analysis, and the fluorescence signals were dominated in the cytoplasm of these two lymphoma cells (Fig. 3C and D). These findings confirmed an easily uptake of ISOIM/siBTK@Exosome by lymphoma cells. Besides, exosome could contribute the uptake of tumor cells for siBTK, as evidenced in Fig. 3E. Specifically, siBTK was labeled by FAM, and then was incubated with SU-DHL-4 and OCL-Ly8 cells for 4 h. Compared to free siBTK, siBTK@Exosome exhibited markedly higher green fluorescence surrounding the nucleus (Fig. 3E), suggesting that the exosome could effectively internalize siBTK into cells. To further discover the underlying uptake mechanism, we pretreated SU-DHL-4 and OCL-Ly8 cells with chlorpromazine, nystatin and colchicine to inhibit clathrin-, caveolin-, and macropinocytosis-mediated endocytosis, respectively. Compared with control (without pretreatment), cells that pretreated by these three inhibitors exhibited reduced uptake for ISOIM/siBTK@Exosome, particularly nystatin (Fig. 3F and G), indicating that ISOIM/siBTK@Exosome was preponderantly internalized by SU-DHL-4 and OCL-Ly8 cells via caveolin-mediated endocytosis. We further intravenously injected PKH26-labeled ISOIM/siBTK@Exosome to OCL-Ly8 cells-bearing mice to observe the tumor targeting of ISOIM/siBTK@Exosome in vivo (Fig. 3H). Fluorescence signals presented at tumor site at 2 h after ISOIM/siBTK@Exosome injection, and were enhanced gradually and reached the maximum at 6h. Fluorescence intensity began to decrease after 12 h of ISOIM/siBTK@Exosome injection. Ex vivo fluorescence imaging was conducted following 48 h of ISOIM/siBTK@Exosome injection. There was a significant enrichment of fluorescence signal in tumor tissue, and weak fluorescence signal in liver and kidney (Fig. 3I). These observations demonstrated the cellular uptake and tumor-targeting ability of ISOIM/siBTK@Exosome.

### 3.3 In vitro anti-lymphoma efficacy

**Fig. 4:**
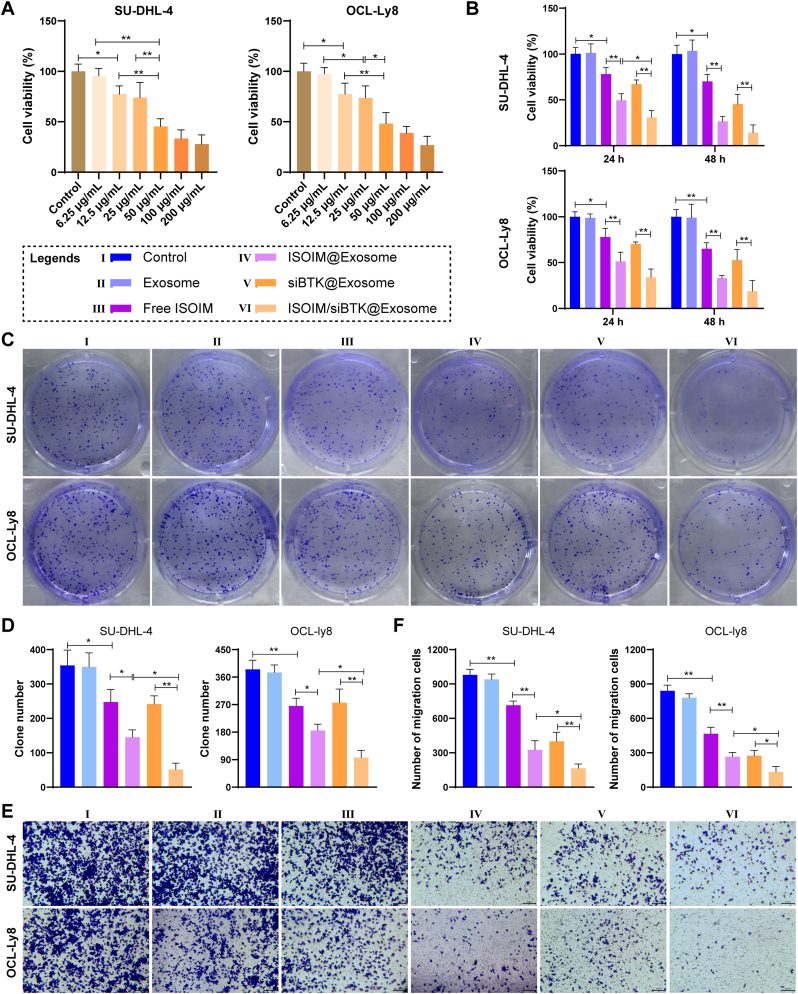
Roles of ISOIM/siBTK@Exosome on proliferation and migration of lymphoma cells. A, Cells viability after treatment with different concentrations of ISOIM/siBTK@Exosome determined by CCK-8; B, Comparisons of cells viability at different treatment groups after 24 h and 48 h of treatment; C-D, colony formation results (C) and corresponding quantitative analysis (D) for evaluating the proliferation of cells at different treatment groups; E-F, representative images (E) and corresponding quantitative analysis (F) for cells migration at different treatment groups. ∗P < 0.05; ∗∗P < 0.01.

**Fig. 5:**
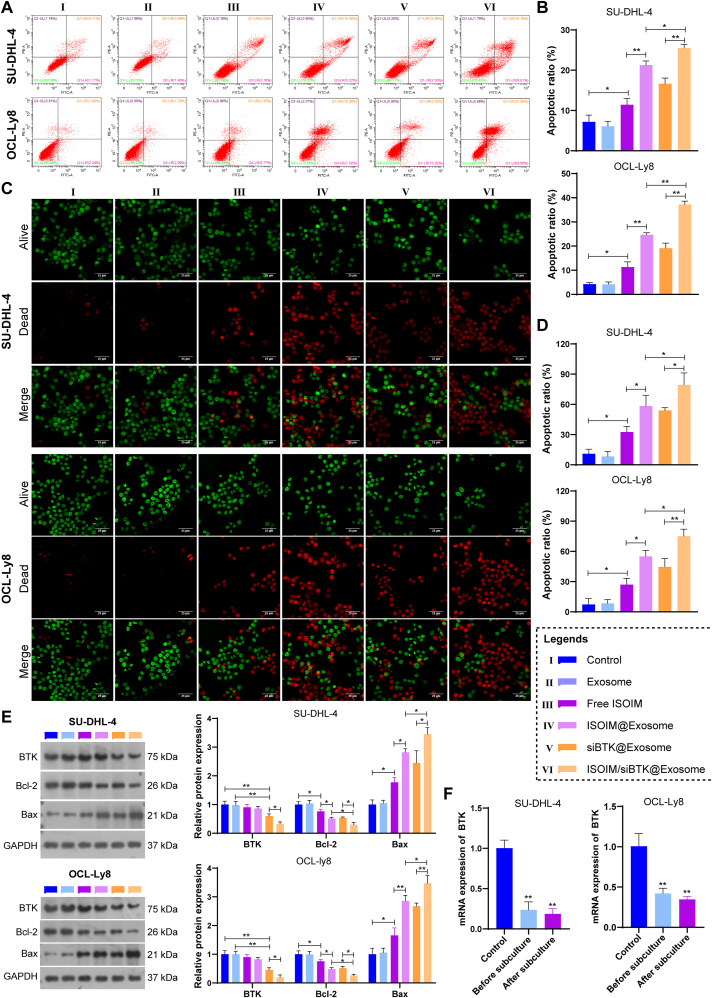
Roles of ISOIM/siBTK@Exosome on apoptosis of lymphoma cells. A-B, Representative images (A) and corresponding quantitative analysis (B) of cells apoptosis determined by FCM analysis; C-D, Representative images (C) and corresponding quantitative analysis (D) of cells survival and death determined by live/dead staining assay; E, Western blot for determining the expression of apoptotic markers Bcl-2 and Bax, as well as the expression of BTK; F, RT-qPCR for revealing the mRNA expression of BTK in treated cells before and after subculture. ∗P < 0.05; ∗∗P < 0.01.

The anti-lymphoma activity of ISOIM/siBTK@Exosome was further demonstrated in vitro. The IC50 of ISOIM/siBTK@Exosome to SU-DHL-4 and OCL-Ly8 cells was measured first, and a dosage of 50 μg/mL ISOIM/siBTK@Exosome reached a half of inhibition for cell viability in both cell lines (Fig. 4A). Hence, 50 μg/mL ISOIM/siBTK@Exosome was selected in following in vitro experiments. Compared to control and empty exosome groups, free ISOIM, ISOIM@Exosome, siBTK@Exosome and ISOIM/siBTK@Exosome exhibited significant repression on cell viability at both 24 and 48 h, particularly the ISOIM/siBTK@Exosome (Fig. 4B). This was further confirmed by colony formation assay, with a lowest clone number after ISOIM/siBTK@Exosome treatment (Fig. 4C and D). Transwell assay indicated that treatment with free ISOIM, ISOIM@Exosome, siBTK@Exosome and ISOIM/siBTK@Exosome could markedly repress the migration of SU-DHL-4 and OCL-Ly8 cells, with the most significant inhibition on cells migration after ISOIM/siBTK@Exosome treatment (Fig. 4E and F). Besides, cells survival was also monitored by Annexin-V/PI and live/dead staining assays. As displayed in Fig. 5A and B, although free ISOIM, ISOIM@Exosome, siBTK@Exosome and ISOIM/siBTK@Exosome treatments significantly stimulated lymphoma apoptotic cell death compared to the control group, the ISOIM/siBTK@Exosome group had the most significant effect. Consistent with this, there presented a highest proportion of dead cells (red) in ISOIM/siBTK@Exosome group in the live/dead staining assay (Fig. 5C and D). Western blot for apoptotic markers expression indicated that apoptosis promotor Bax expression elevated whereas apoptosis repressor BCL-2 reduced after free ISOIM, ISOIM@Exosome, siBTK@Exosome and ISOIM/siBTK@Exosome treatments, particularly in ISOIM/siBTK@Exosome group (Fig. 5E). These findings suggested that ISOIM/siBTK@Exosome could inhibit proliferation and migration, while induce apoptotic death of lymphoma cells. Additionally, expression of BTK was also detected. With the aids of exosome, si-BTK (siBTK@Exosome and ISOIM/siBTK@Exosome) markedly decreased the expression of BTK in both SU-DHL-4 and OCL-Ly8 cells (Fig. 5E), and such decrease on BTK expression was sustained and stable following three times of subculture (Fig. 5F).

### 3.4 T cells activation and macrophage polarization in vitro

**Fig. 6:**
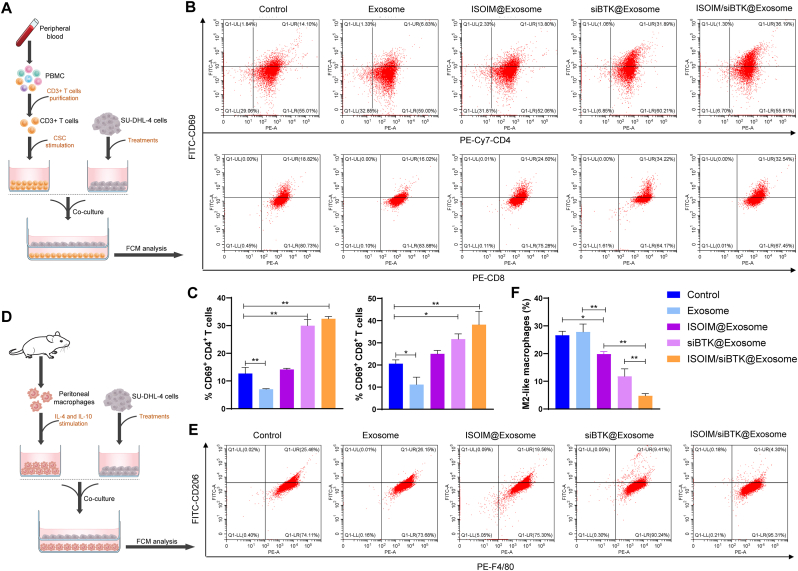
Roles of ISOIM/siBTK@Exosome on T cells activation and macrophage polarization. A, Experimental scheme for the co-culture of stimulated CD3^+^ T cells and treated SU-DHL-4 cells; B-C, Representative images (B) and corresponding quantitative analysis (C) showing the percentages of CD69^+^CD4^+^ T cells and CD69^+^CD8^+^ T cells in the coculture system; D, Experimental scheme for the co-culture of stimulated peritoneal macrophages and treated SU-DHL-4 cells; E-F, Representative images (E) and corresponding quantitative analysis (F) showing the percentages of M2 macrophages in the coculture system. ∗P < 0.05; ∗∗P < 0.01.

To examine the functions of ISOIM/siBTK@Exosome in T‐cell activation, CD3^+^ T cells were isoloated and were stimulated by CSC, and then were co-incubated with treated SU-DHL-4 cells for 24 h (Fig. 6A). The percentages of CD69^+^CD4^+^ T cells and CD69^+^CD8^+^ T cells were markedly elevated in siBTK@Exosome and ISOIM/siBTK@Exosome groups compared to empty exosome group (Fig. 6B and C), indicating that siBTK@Exosome and ISOIM/siBTK@Exosome might contribute the T cells activation in vitro. We further assessed the action of ISOIM/siBTK@Exosome on macrophage polarization in vitro, which was conducted by co-incubation of macrophages and treated SU-DHL-4 cells (Fig. 6D). FCM analysis exhibited that there was an obvious decrease of M2 macrophages in ISOIM@Exosome, siBTK@Exosome and ISOIM/siBTK@Exosome group, particularly in the ISOIM/siBTK@Exosome group (Fig. 6E and F), suggesting the action of ISOIM/siBTK@Exosome in triggering macrophages polarization to an anti-tumor phenotype.

### 3.5 In vivo anti-lymphoma activity and security assessment of ISOIM/siBTK@Exosome

**Fig. 7:**
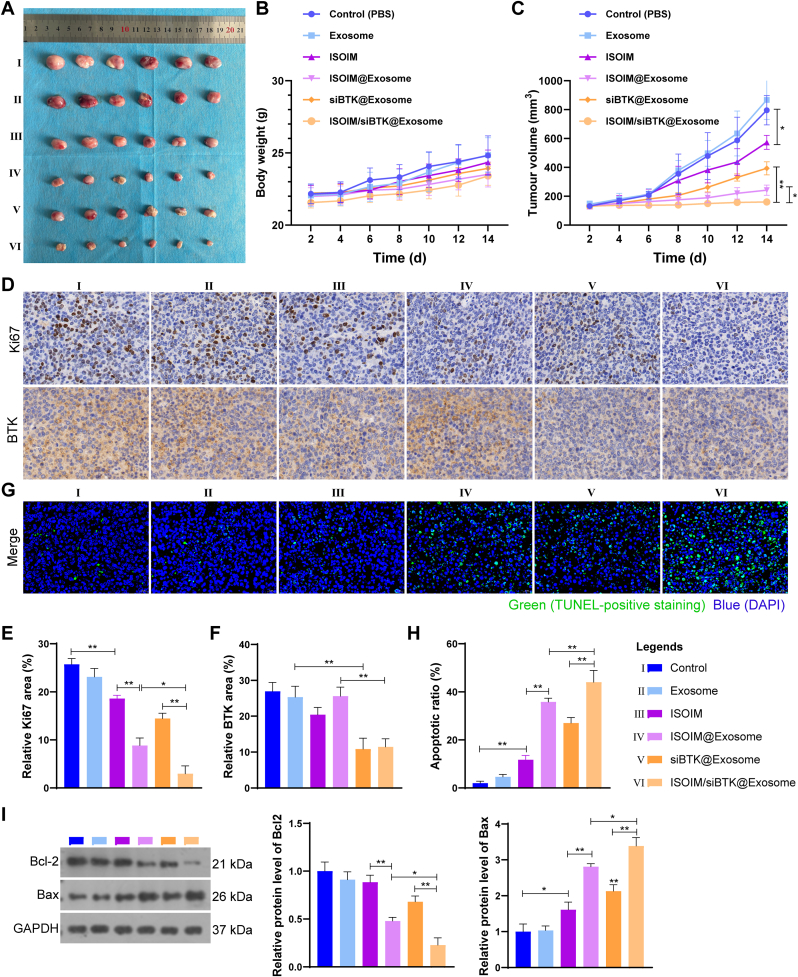
Evaluation of anti-tumor activity in vivo. A, Images of the dissected tumor tissue from tumor-bearing mice in each group (n = 6); B-C, Body weight (B) and tumor volume (C) of mice in each group; D-F, Representative images (D) and corresponding quantitative analysis of Ki67 (E) and BTK (F) immumohistochemical staining in tumor tissue; G-H, Representative images (G) and corresponding quantitative analysis of apoptosis (H) in tumor tissue determined by TUNEL staining; I, Western blot analysis of apoptotic markers Bcl-2 and Bax expression in tumor tissue. ∗P < 0.05; ∗∗P < 0.01.

The anti-lymphoma activity of ISOIM/siBTK@Exosome in vivo was further verified in OCL-Ly8 cells-bearing mice. Compared to control and empty exosome groups, the tumor volume of mice in other groups were smaller, particularly smallest in mice treated with ISOIM/siBTK@Exosome (Fig. 7A–C). Consistent with this, we observed a lowest expression of proliferation marker Ki67 in tumor tissue of mice after ISOIM/siBTK@Exosome treatment (Fig. 7D and E). Besides, the reduced expression of BTK in siBTK@Exosome and ISOIM/siBTK@Exosome groups also demonstrated that exosomes successfully delivered drugs to tumor tissues for therapeutic effect (Fig. 7D and F). We further conducted TUNEL staining on tumor tissues from mice in each group. As expected, compared to control and empty exosome groups, there was significant apoptotic tumor injury in other treatment group, especially ISOIM/siBTK@Exosome group (Fig. 7G and H), which was also confirmed by the expression of apoptotic markers Bcl-2 and Bax in tumor tissue (Fig. 7I). Altogether, these results indicated outstanding anti-lymphoma activity of ISOIM/siBTK@Exosome in vivo.

**Fig. 8:**
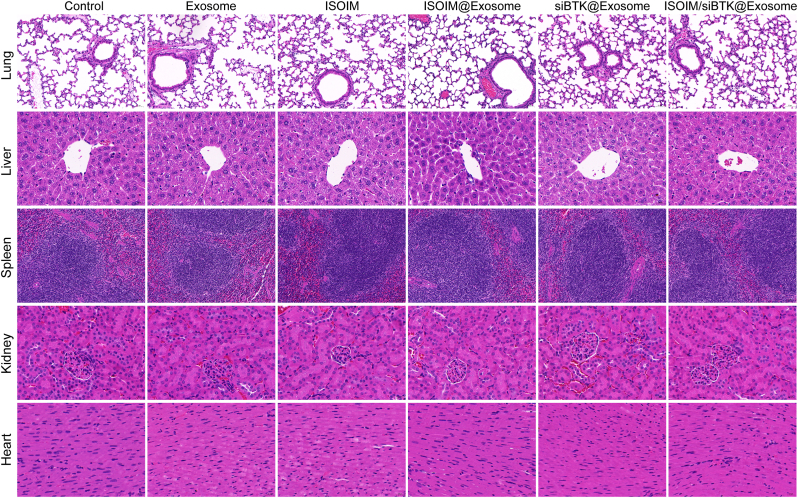
Biosafety of ISOIM/siBTK@Exosome. Representative H&E staining images of major organs (heart, liver, spleen, lung and kidney) from tumor-bearing mice in each group.

Additionally, we also assessed the potential toxicity of ISOIM/siBTK@Exosome by means of H&E staining on the main organ tissues. No obvious injury was observed on major organ tissue of mice in each treatment group, consistently with mice in control and empty exosome groups (Fig. 8). Therefore, we believe that the established ISOIM/siBTK@Exosome is biosafe.

## 4 Discussion

Recently, with the development of synthetic chemistry technology, therapeutic nanoparticles for the drug delivery have shown an ascending trend. Emerging drug delivery systems based on liposomes, polymers, and nanoparticles have been used to improve the solubility, stability, and efficacy of drug chemical and biological functions [[35], [36], [37], [38], [39]]. However, there are still some challenges with these nanocarriers. For example, the nanocarriers and their degradation products might be cytotoxic (more focus on biosafety and toxicity is required), and can be quickly cleared by mononuclear phagocytic system [[40], [41], [42]]. Only a small number of nanocarriers are currently approved for clinical use. To address the limitations of conventional delivery systems, more and more research is focused on developing more effective and safe carriers of endogenous cellular or subcellular structures, such as exosomes. Therefore, exosomes have been advantageous for the delivery application [43].

Exosomes as drug delivery systems have the following advantages. Firstly, exosomes are vesicles produced by cells, which inherit the same phospholipid membrane of cell membrane. The exosomes have the potential to internalize in cells through endocytosis [44,45], that is beneficial for the delivery of cargoes. Secondly, exosomes show high uptake efficiency through direct interaction with specific extracellular proteins or direct membrane fusion or endocytosis of target cells mediated by integrins from specific sources [46,47]. Thirdly, exosomes are not easily cleared by the peripheral mononuclear phagocytosis system because the existence of integrin-associated transmembrane proteins. For example, CD47 protein, a "don't eat me" signal, prevents exosomes against from phagocytosis by immune cells [[48], [49], [50]]. Fourthly, in addition to higher biocompatibility and lower toxicity and immunogenicity, exosomes have a high tumor homing ability, which enables exosomes being more effective in delivering cancer therapeutics [47,51]. In this study, lymphoma cell-derived exosomes were selected for the delivery of si-BTK and ISOIM. Tumor cell-derived exosomes are a suitable drug delivery system for tumor therapy because of their ability to successfully target tumor cells due to the homotypic features with tumor cells, and be effectively absorbed by tumor cells [46,52]. Multiple studies have successfully used tumor-cell-derived exosomes to deliver chemotherapy or other factors [[53], [54], [55]]. For example, based on exosomes derived from cancer cells, Qiao et al. [46] prepared Doxil-loaded engineered exosomes to examine their tumor targeting and anti-tumor activity. They found that the engineered exosomes preferentially fused with their parent cancer cells in vitro, and exhibited better tumor targeting and longer retention in tumor tissue compared to Doxil alone in vivo [46].

Loading drugs into exosomes is one of the key steps in developing exosome-based drug delivery systems, and following major factors should be considered: how to achieve better encapsulation or loading efficiency; how to maintain the structural integrity of the exosomes; and how to maintain the activity of the drugs [56,57]. In our research, we used electroporation to load si-BTK and ISOIM into exosomes. Electroporation is the process of forming hydrophilic pores by applying an external electric field, which can instantaneously increase the permeability of exosome membranes by forming "temporary channels" to load chemicals, DNA, RNA, or drugs into the exosomes [57,58]. After the drug is loaded into the exosome through temporary pores, the integrity of the exosome membrane is quickly restored. Studies have demonstrated that exogenous oligonucleotides, for instance, siRNA, can be effectively encapsulated into exosomes by means of electroporation, which protects siRNA against from degradation and contribute the intact delivery of siRNA to target cells [59]. The decrease of BTK expression in cells and tumor tissue after siBTK@Exosome and ISOIM/siBTK@Exosome treatments confirmed the intact delivery of si-BTK to lymphoma cells. The loading capacity of si-BTK and ISOIM in exosomes was determined to be approximately 11.23 % and 13.01 %, respectively. Although the drug load of exosomes is slightly worse than that of liposomes, the lipid bilayer membrane of exosomes gives them stronger protection against drug loading. The drug release assessment confirmed that release rate of ISOIM and si-BTK reached about 80 % at 48 h.

The therapeutic effect of the prepared ISOIM/siBTK@Exosome was evaluated in two tumor cell lines and in tumor-bearing mice. ISOIM/siBTK@Exosome exhibited significant repression on cell growth and migration, while markedly induced apoptotic cell death in vitro when compared to free ISOIM, ISOIM@Exosome and siBTK@Exosome, respectively. Similar results were observed in tumor-bearing mice. These findings suggested that the prepared ISOIM/siBTK@Exosome had excellent anti-lymphoma efficacy, which might be explained by the excellent tumor targeting of exosome as carriers and the synergistic therapeutic roles of the combination of ISOIM and si-BTK. Besides, ISOIM-loaded exosomes also reduced the accumulation of ISOIM in non-target organs and prevented tissue toxicity from off-target. This was confirmed by the security assessment in H&E staining. The underlying molecular mechanisms were preliminarily explored. ISOIM has been reported to inhibit tumor by regulating epithelial-mesenchymal transformation (EMT) [30], and EMT has been demonstrated to contribute to tumor immune escape. BTK was identified as a key regulatory molecule of tumor-associated inflammation, in which BTK inhibition led to an increased presence of tumor-associated CD8^+^ T cells associated with inhibition of tumor growth [60]. Ibrutinib, a BTK inhibitor, differentially regulates LPS-mediated surface marker expression and cytokines production in dendritic cells to enhance CD4^+^ T cell activation [61]. Therefore, we hypothesize that the prepared ISOIM/siBTK@Exosome can regulate immune escape. Macrophages are among the main components of the DLBCL microenvironment [62], and there are close connections between macrophages infiltration and poor prognosis of DLBCL patients after R-CHOP therapy [63,64]. Patients with DLBCL showed lymphocytopenia in the CD4^+^ T, CD8^+^ T, and NK cell subpopulations, and an increased number of activated memory CD4^+^ T cells in the infiltrating area of B-cell lymphoma was associated with a decreased rate of cell proliferation [65]. Therefore, we examined the functions of ISOIM/siBTK@Exosome in T‐cell activation and macrophage polarization in vitro. There was an obvious increase of CD69^+^CD4^+^ T cells and CD69^+^CD8^+^ T cells, and an obvious decrease of M2 macrophages after ISOIM/siBTK@Exosome treatment, suggested that ISOIM/siBTK@Exosome could promote T cells activation and prevent macrophage M2 polarization. However, the underlying mechanism of ISOIM/siBTK@Exosome regulating T cells activation and macrophage polarization remains unclear, and will be investigated in our further studies. We believe that this will help us have a more comprehensive understanding of the therapeutic potential of ISOIM/siBTK@Exosome in DLBCL.

Furthermore, the use of tumor-derived exosomes as delivery vehicles presents a compelling opportunity for personalized cancer therapy. These exosomes, by retaining the surface markers and membrane proteins of their parent tumor cells, demonstrate superior targeting ability, enabling efficient delivery of therapeutic agents directly to the tumor microenvironment. This natural homing capability enhances the specificity of drug accumulation at tumor sites, thereby maximizing efficacy while minimizing systemic toxicity. By combining si-BTK with ISOIM in a single exosomal platform, this strategy not only delivers a targeted genetic knockdown of BTK but also exploits the pharmacological benefits of a bioactive natural compound, creating a potent synergistic anti-lymphoma effect. Additionally, the dual-action approach of ISOIM/siBTK@Exosome extends beyond cytotoxic effects to include modulation of the tumor immune microenvironment. The observed activation of CD4^+^ and CD8^+^ T cells, along with the suppression of M2 macrophage polarization, suggests that this therapeutic system may help reverse immune suppression commonly found in DLBCL. These immunomodulatory effects are crucial, as they contribute to sustained tumor suppression and may enhance the efficacy of subsequent or concurrent immunotherapies. Altogether, the findings support ISOIM/siBTK@Exosome as a promising and versatile strategy for improving the outcomes of patients with DLBCL through both direct tumor inhibition and immune system engagement.

While our study highlights the promising in vitro immunomodulatory potential of ISOIM/siBTK@Exosome, further in vivo validation remains essential. Specifically, comprehensive flow cytometry analyses of tumor-infiltrating immune cells in treated mice would provide direct evidence of T cell activation and macrophage polarization within the tumor microenvironment. Such experiments could clarify whether the observed in vitro effects translate into effective reprogramming of the tumor immune landscape in vivo. These future studies will be critical to establish ISOIM/siBTK@Exosome not only as a direct anti-lymphoma therapeutic but also as an immune-modulating nanoplatform capable of synergistically engaging the host immune system. Future studies should focus on quantitatively confirming the synergistic effects of ISOIM and siBTK by applying the Combination Index (CI) method. Such analysis will allow a precise determination of whether their combined therapeutic impact is synergistic, additive, or antagonistic, thereby providing deeper mechanistic insights and guiding potential dose optimization for clinical translation. Given that BTK inhibitors are clinically established in MCL and CLL but not in DLBCL, future studies should evaluate the therapeutic efficacy of ISOIM/siBTK@Exosome in MCL and CLL models. Such validation would not only broaden the scope of application but also bridge our findings more directly to existing clinical contexts where BTK inhibition has already proven beneficial.

## 5 Conclusion

In this study, we proposed cancer exosome-based drug delivery system for the combination therapy of si-BTK and ISOIM for DLBCL. The ISOIM/siBTK@Exosome was a highly effective and non-toxic therapeutic strategy in DLBCL, which exhibited the advantages in active targeting of lymphoma cells, inhibiting immune escape and high safety. Targeted delivery of si-BTK overcomes the acquired resistance to current BTK inhibitors as well as corresponding toxic side effects. Meanwhile, the combination of ISOIM and si-BTK provided dual anti-lymphoma effects. In summary, our current study indicates that ISOIM/siBTK@Exosome may provide novel targeted therapeutic strategy to be applied in the clinical management of patients with DLBCL, which holds excellent promise for improving the clinical outcomes of DLBCL.

## CRediT authorship contribution statement

**Ruowen Sun:** Writing – review & editing, Writing – original draft, Supervision, Software, Formal analysis, Data curation. **Yanchao Yang:** Project administration, Methodology, Investigation, Formal analysis, Data curation. **Bin Zhang:** Software, Resources, Project administration, Methodology. **Jiayuan Chen:** Validation, Supervision, Software, Resources, Project administration. **Ye Wang:** Project administration, Methodology, Investigation, Funding acquisition, Formal analysis, Data curation. **Zehui Jiang:** Validation, Supervision, Software, Resources, Project administration. **Linlin Zhang:** Methodology, Investigation, Funding acquisition, Formal analysis, Data curation. **Milad Ashrafizadeh:** Writing – review & editing, Writing – original draft. **Gautam Sethi:** Writing – review & editing, Writing – original draft. **Jing Shen:** Project administration, Methodology, Investigation, Formal analysis, Data curation, Conceptualization. **Zuofei Chi:** Supervision, Software, Resources, Project administration, Formal analysis, Data curation, Conceptualization.

## Ethics approval

All animal experiments were approved by the Institutional Animal Care and Use Committee of YANGZHOU UNIVERSITY (Approval No. 202410028), and conducted in accordance with ARRIVE guidelines.

## Funding

Not applicable.

## Declaration of competing interest

The authors declare that they have no known competing financial interests or personal relationships that could have appeared to influence the work reported in this paper.

## Data Availability

Data will be made available on request.

## References

[1] Armitage J.O., Gascoyne R.D., Lunning M.A., Cavalli F. (2017). Non-Hodgkin lymphoma. Lancet (London, England).

[2] Bray F., Laversanne M., Sung H., Ferlay J., Siegel R.L., Soerjomataram I., Jemal A. (2024). Global cancer statistics 2022: GLOBOCAN estimates of incidence and mortality worldwide for 36 cancers in 185 countries. Ca - Cancer J. Clin..

[3] Liu W., Liu J., Song Y., Wang X., Mi L., Cai C., Zhao D., Wang L., Ma J., Zhu J. (2022). Burden of lymphoma in China, 1990-2019: an analysis of the global burden of diseases, injuries, and risk factors study 2019. Aging.

[4] Barraclough A., Hawkes E., Sehn L.H., Smith S.M. (2024). Diffuse large B-cell lymphoma. Hematol. Oncol..

[5] Wallace D.S., Loh K.P., Casulo C. (2025). How I treat older patients with relapsed/refractory diffuse large B-cell lymphoma. Blood.

[6] Ren W., Wan H., Own S.A., Berglund M., Wang X., Yang M., Li X., Liu D., Ye X., Sonnevi K. (2024). Genetic and transcriptomic analyses of diffuse large B-cell lymphoma patients with poor outcomes within two years of diagnosis. Leukemia.

[7] Wilson W.H., Young R.M., Schmitz R., Yang Y., Pittaluga S., Wright G., Lih C.J., Williams P.M., Shaffer A.L., Gerecitano J. (2015). Targeting B cell receptor signaling with ibrutinib in diffuse large B cell lymphoma. Nat. Med..

[8] Wang W.T., Xing T.Y., Du K.X., Hua W., Guo J.R., Duan Z.W., Wu Y.F., Wu J.Z., Li Y., Yin H. (2024). CD30 protects EBV-positive diffuse large B-cell lymphoma cells against mitochondrial dysfunction through BNIP3-mediated mitophagy. Cancer Lett..

[9] Valla K., Flowers C.R., Koff J.L. (2018). Targeting the B cell receptor pathway in non-Hodgkin lymphoma. Expet Opin. Invest. Drugs.

[10] Dunleavy K., Erdmann T., Lenz G. (2018). Targeting the B-cell receptor pathway in diffuse large B-cell lymphoma. Cancer Treat Rev..

[11] Pal Singh S., Dammeijer F., Hendriks R.W. (2018). Role of Bruton's tyrosine kinase in B cells and malignancies. Mol. Cancer.

[12] Shirley M. (2022). Bruton tyrosine kinase inhibitors in B-cell malignancies: their use and differential features. Targeted Oncol..

[13] Rozkiewicz D., Hermanowicz J.M., Kwiatkowska I., Krupa A., Pawlak D. (2023). Bruton's tyrosine kinase inhibitors (BTKIs): review of preclinical studies and evaluation of clinical trials. Molecules.

[14] Huang S., Pan J., Jin J., Li C., Li X., Huang J., Huang X., Yan X., Li F., Yu M. (2019). Abivertinib, a novel BTK inhibitor: anti-Leukemia effects and synergistic efficacy with homoharringtonine in acute myeloid leukemia. Cancer Lett..

[15] Sibaud V., Beylot-Barry M., Protin C., Vigarios E., Recher C., Ysebaert L. (2020). Dermatological toxicities of Bruton's tyrosine kinase inhibitors. Am. J. Clin. Dermatol..

[16] Wang E., Mi X., Thompson M.C., Montoya S., Notti R.Q., Afaghani J., Durham B.H., Penson A., Witkowski M.T., Lu S.X. (2022). Mechanisms of resistance to noncovalent Bruton's tyrosine kinase inhibitors. N. Engl. J. Med..

[17] Dastgerdi N.K., Dastgerdi N.K., Bayraktutan H., Costabile G., Atyabi F., Dinarvand R., Longobardi G., Alexander C., Conte C. (2024). Enhancing siRNA cancer therapy: multifaceted strategies with lipid and polymer-based carrier systems. Int. J. Pharm..

[18] Wang T., Shigdar S., Shamaileh H.A., Gantier M.P., Yin W., Xiang D., Wang L., Zhou S.F., Hou Y., Wang P. (2017). Challenges and opportunities for siRNA-based cancer treatment. Cancer Lett..

[19] Moazzam M., Zhang M., Hussain A., Yu X., Huang J., Huang Y. (2024). The landscape of nanoparticle-based siRNA delivery and therapeutic development. Mol. Ther..

[20] Pérez-Carrión M.D., Posadas I., Ceña V. (2024). Nanoparticles and siRNA: a new era in therapeutics?. Pharmacol. Res..

[21] Ye J., Li D., Jie Y., Luo H., Zhang W., Qiu C. (2024). Exosome-based nanoparticles and cancer immunotherapy. Biomed. Pharmacother..

[22] Liang Y., Duan L., Lu J., Xia J. (2021). Engineering exosomes for targeted drug delivery. Theranostics.

[23] Kimiz-Gebologlu I., Oncel S.S. (2022). Exosomes: large-scale production, isolation, drug loading efficiency, and biodistribution and uptake. J. Contr. Release.

[24] García-Fernández J., Fuente Freire M. (2023). Exosome-like systems: nanotechnology to overcome challenges for targeted cancer therapies. Cancer Lett..

[25] Lee J.H., Song J., Kim I.G., You G., Kim H., Ahn J.-H., Mok H. (2022). Exosome-mediated delivery of transforming growth factor-β receptor 1 kinase inhibitors and toll-like receptor 7/8 agonists for combination therapy of tumors. Acta Biomater..

[26] Mondal S., Bandyopadhyay S., Ghosh M.K., Mukhopadhyay S., Roy S., Mandal C. (2012). Natural products: promising resources for cancer drug discovery. Anti Cancer Agents Med. Chem..

[27] Lai Y., Han T., Zhan S., Jiang Y., Liu X., Li G. (2021). Antiviral activity of isoimperatorin against influenza A virus in vitro and its inhibition of neuraminidase. Front. Pharmacol..

[28] Xu Y., Xia D., Deng S., Liang M. (2025). Isoimperatorin inhibits angiogenesis by suppressing VEGFR2 signaling pathway. Cardiovasc. Drugs Ther..

[29] Fan L., Li Z., Gao L., Zhang N., Chang W. (2023). Isoimperatorin alleviates lipopolysaccharide-induced periodontitis by downregulating ERK1/2 and NF-κB pathways. Open Life Sci..

[30] Kim N.Y., Jung Y.Y., Yang M.H., Um J.Y., Sethi G., Ahn K.S. (2022). Isoimperatorin down-regulates epithelial mesenchymal transition through modulating NF-κB signaling and CXCR4 expression in colorectal and hepatocellular carcinoma cells. Cell. Signal..

[31] Tong K., Xin C., Chen W. (2017). Isoimperatorin induces apoptosis of the SGC-7901 human gastric cancer cell line via the mitochondria-mediated pathway. Oncol. Lett..

[32] Yang H.B., Gao H.R., Ren Y.J., Fang F.X., Tian H.T., Gao Z.J., Song W., Huang S.M., Zhao A.F. (2018). Effects of isoimperatorin on proliferation and apoptosis of human gastric carcinoma cells. Oncol. Lett..

[33] Ko H.J., Park S.Y., Sim D.Y., Kim S.H., Hur S., Lee J.H., Kim Y. (2024). Apoptotic effect of isoimpertorin via inhibition of c-Myc and SIRT1 signaling Axis. Int. J. Mol. Sci..

[34] Zhao Q., Sun X., Wu B., Shang Y., Huang X., Dong H., Liu H., Chen W., Gui R., Li J. (2021). Construction of homologous cancer cell membrane camouflage in a nano-drug delivery system for the treatment of lymphoma. J. Nanobiotechnol..

[35] Park K. (2014). Controlled drug delivery systems: past forward and future back. J. Contr. Release.

[36] Allen T.M., Cullis P.R. (2013). Liposomal drug delivery systems: from concept to clinical applications. Adv. Drug Deliv. Rev..

[37] Manzari M.T., Shamay Y., Kiguchi H., Rosen N., Scaltriti M., Heller D.A. (2021). Targeted drug delivery strategies for precision medicines. Nat. Rev. Mater..

[38] Nance E., Pun S.H., Saigal R., Sellers D.L. (2022). Drug delivery to the central nervous system. Nat. Rev. Mater..

[39] Wu D., Chen Q., Chen X., Han F., Chen Z., Wang Y. (2023). The blood–brain barrier: structure, regulation and drug delivery. Signal Transduct. Targeted Ther..

[40] Najahi-Missaoui W., Arnold R.D., Cummings B.S. (2020). Safe nanoparticles: are we there yet?. Int. J. Mol. Sci..

[41] Storm G., Belliot S.O., Daemen T., Lasic D.D. (1995). Surface modification of nanoparticles to oppose uptake by the mononuclear phagocyte system. Adv. Drug Deliv. Rev..

[42] García K.P., Zarschler K., Barbaro L., Barreto J.A., O'Malley W., Spiccia L., Stephan H., Graham B. (2014). Zwitterionic‐coated “stealth” nanoparticles for biomedical applications: recent advances in countering biomolecular corona formation and uptake by the mononuclear phagocyte system. Small.

[43] Sadeghi S., Tehrani F.R., Tahmasebi S., Shafiee A., Hashemi S.M. (2023). Exosome engineering in cell therapy and drug delivery. Inflammopharmacology.

[44] Herrmann I.K., Wood M.J.A., Fuhrmann G. (2021). Extracellular vesicles as a next-generation drug delivery platform. Nat. Nanotechnol..

[45] Kim Y.K., Choi Y., Nam G.H., Kim I.S. (2020). Functionalized exosome harboring bioactive molecules for cancer therapy. Cancer Lett..

[46] Qiao L., Hu S., Huang K., Su T., Li Z., Vandergriff A., Cores J., Dinh P.U., Allen T., Shen D. (2020). Tumor cell-derived exosomes home to their cells of origin and can be used as Trojan horses to deliver cancer drugs. Theranostics.

[47] Shao J., Zaro J., Shen Y. (2020). Advances in exosome-based drug delivery and tumor targeting: from tissue distribution to intracellular fate. Int. J. Nanomed..

[48] Jaiswal S., Jamieson C.H., Pang W.W., Park C.Y., Chao M.P., Majeti R., Traver D., van Rooijen N., Weissman I.L. (2009). CD47 is upregulated on circulating hematopoietic stem cells and leukemia cells to avoid phagocytosis. Cell.

[49] Koh E., Lee E.J., Nam G.H., Hong Y., Cho E., Yang Y., Kim I.S. (2017). Exosome-SIRPα, a CD47 blockade increases cancer cell phagocytosis. Biomaterials.

[50] Shimizu A., Sawada K., Kobayashi M., Yamamoto M., Yagi T., Kinose Y., Kodama M., Hashimoto K., Kimura T. (2021). Exosomal CD47 plays an essential role in immune evasion in ovarian cancer. Mol. Cancer Res..

[51] Du S., Guan Y., Xie A., Yan Z., Gao S., Li W., Rao L., Chen X., Chen T. (2023). Extracellular vesicles: a rising star for therapeutics and drug delivery. J. Nanobiotechnol..

[52] Du S., Qian J., Tan S., Li W., Liu P., Zhao J., Zeng Y., Xu L., Wang Z., Cai J. (2022). Tumor cell-derived exosomes deliver TIE2 protein to macrophages to promote angiogenesis in cervical cancer. Cancer Lett..

[53] Wang J., Zhu X., Jiang H., Ji M., Wu Y., Chen J. (2024). Cancer cell-derived exosome based dual-targeted drug delivery system for non-small cell lung cancer therapy. Colloids Surf. B Biointerfaces.

[54] Li M., Yin S., Xu A., Kang L., Ma Z., Liu F., Yang T., Sun P., Tang Y. (2023). Synergistic phototherapy-molecular targeted therapy combined with tumor exosome nanoparticles for oral squamous cell carcinoma treatment. Pharmaceutics.

[55] Yong T., Zhang X., Bie N., Zhang H., Zhang X., Li F., Hakeem A., Hu J., Gan L., Santos H.A. (2019). Tumor exosome-based nanoparticles are efficient drug carriers for chemotherapy. Nat. Commun..

[56] Xi X.M., Xia S.J., Lu R. (2021). Drug loading techniques for exosome-based drug delivery systems. Pharmazie.

[57] Zeng H., Guo S., Ren X., Wu Z., Liu S., Yao X. (2023). Current strategies for exosome cargo loading and targeting delivery. Cells.

[58] Kumar P., Nagarajan A., Uchil P.D. (2019).

[59] Wu X., Ban C., Deng W., Bao X., Tang N., Wu Y., Deng Z., Xiong J., Zhao Q. (2024). Unveiling the PDK4-centered rituximab-resistant mechanism in DLBCL: the potential of the "Smart" exosome nanoparticle therapy. Mol. Cancer.

[60] Taylor M.H., Betts C.B., Maloney L., Nadler E., Algazi A., Guarino M.J., Nemunaitis J., Jimeno A., Patel P., Munugalavadla V. (2022). Safety and efficacy of pembrolizumab in combination with acalabrutinib in advanced head and neck squamous cell carcinoma: phase 2 proof-of-concept study. Clin. Cancer Res..

[61] Natarajan G., Oghumu S., Terrazas C., Varikuti S., Byrd J.C., Satoskar A.R. (2016). A Tec kinase BTK inhibitor ibrutinib promotes maturation and activation of dendritic cells. OncoImmunology.

[62] Liu M., Bertolazzi G., Sridhar S., Lee R.X., Jaynes P., Mulder K., Syn N., Hoppe M.M., Fan S., Peng Y. (2024). Spatially-resolved transcriptomics reveal macrophage heterogeneity and prognostic significance in diffuse large B-cell lymphoma. Nat. Commun..

[63] Li Y.L., Shi Z.H., Wang X., Gu K.S., Zhai Z.M. (2019). Tumor-associated macrophages predict prognosis in diffuse large B-cell lymphoma and correlation with peripheral absolute monocyte count. BMC Cancer.

[64] Riihijärvi S., Fiskvik I., Taskinen M., Vajavaara H., Tikkala M., Yri O., Karjalainen-Lindsberg M.L., Delabie J., Smeland E., Holte H. (2015). Prognostic influence of macrophages in patients with diffuse large B-cell lymphoma: a correlative study from a Nordic phase II trial. Haematologica.

[65] Tamma R., Ranieri G., Ingravallo G., Annese T., Oranger A., Gaudio F., Musto P., Specchia G., Ribatti D. (2020). Inflammatory cells in diffuse large B cell lymphoma. J. Clin. Med..

